# Case Report: Late-onset BK polyomavirus-associated nephropathy in kidney transplant recipients: two cases and insights into underlying mechanisms

**DOI:** 10.3389/fmed.2026.1799421

**Published:** 2026-07-06

**Authors:** Tanja Belčič Mikič, Matic Bošnjak, Miha Arnol

**Affiliations:** 1 Department of Nephology, University Medical Center Ljubljana, Ljubljana, Slovenia; 2 Faculty of Medicine, University of Ljubljana, Ljubljana, Slovenia; 3 Faculty of Medicine, Institute of Pathology, University of Ljubljana, Ljubljana, Slovenia

**Keywords:** BK polyomavirus-associated nephropathy, diagnosis, immunosuppression, kidney transplantation, pathophysiology

## Abstract

BK polyomavirus–associated nephropathy (BKPyVAN) results from primary infection or reactivation of BK polyomavirus (BKPyV) and remains a significant complication in kidney transplant recipients, contributing to allograft dysfunction and premature allograft loss. Most cases occur within the first 2 years post-transplant, when cell-mediated immunity is most suppressed due to induction immunosuppression. Here, we describe two unusual cases of late-onset BKPyVAN occurring in the absence of intensified immunosuppression and provide a review of potential mechanisms underlying its development in kidney transplant recipients. In the first patient, BKPyVAN developed 9 years after transplantation during maintenance dual immunosuppression with tacrolimus and mycophenolic acid, without prior rejection episodes or exposure to intensified immunosuppression. In the second patient, BKPyVAN first occurred 3 years post-transplant in the setting of concomitant cytomegalovirus colitis, also during dual immunosuppression with tacrolimus and mycophenolic acid. Despite reduction of immunosuppression, BKPyVAN persisted at 1-year follow-up and was associated with a gradual decline in allograft function. These cases highlight that late-onset BKPyVAN may develop even without intensified immunosuppression and can lead to significant allograft injury. Improved strategies are needed to identify patients at risk for late-onset BKPyVAN and to optimize therapeutic management.

## 1 Introduction

BK polyomavirus-associated nephropathy (BKPyVAN) is caused by infection with, or reactivation of, BK polyomavirus (BKPyV) and is one of the most significant opportunistic viral infections following transplantation (1). In a recent single-center retrospective study, BKPyVAN occurred in 32% of kidney transplant recipients with opportunistic infections in the first year, and in 18% of patients more than 1 year after transplantation (2). BKPyV is a small, double-stranded DNA virus first discovered in 1970 in London, England, in a kidney transplant recipient who presented with a ureteric stricture requiring surgical correction (3). It was named after the initials of the first patient from whom it was isolated (4). Its genome consists of three regions: an early coding region, a late coding region, and a non-coding control region (NCCR) (5). The early coding region contains genes encoding the large and small tumor antigens (TAg and tAg), while the late coding region encodes the capsid proteins VP1–3 and agnoprotein, which are involved in virion assembly, maturation, and release (6). The NCCR is the site of transcriptional control and displays hypervariability due to rearrangements and single nucleotide polymorphisms (SNPs) (7). BKPyV genotypes include Ia, Ib1, Ib2, Ic, II, III, IVb1, and IVc2, representing five distinct serotypes, as BKPyV-Ib1 and BKPyV-Ib2 have unique cellular interactions (5, 8). The most prevalent genotype worldwide is genotype I, followed by genotype IV (9).

BKPyV infection is considered ubiquitous in the general population and is typically acquired asymptomatically during childhood, with high seroprevalence rates in adults of nearly 90% (10). The primary routes of transmission are the oral, gastrointestinal, and respiratory mucosa. BKPyV TAg expression and viral production depend on the host initially entering the S phase, and BKPyV primarily replicates during host re-replication (11). After a primary BKPyV DNAemia, BKPyV persists in the kidney and uroepithelial cells, resulting in lifelong infection (12). In the context of immunosuppression, loss of immune control leads to increased viral replication (13). If the virus replicates sufficiently, it can cross peritubular capillaries and cause viremia, followed by invasion of the kidney allograft, recognized as BKPyVAN (14).

BKPyV-related complications are most common in the first year after transplantation, when net immunosuppression is highest due to induction therapy (1, 2). BKPyV DNAuria is the earliest manifestation of the disease, detected in up to 40% of kidney transplant recipients in the first-year after transplantation (1). BKPyV DNAemia can develop in about one-third of patients with persistent BKPyV DNAuria over a period of 2–6 weeks and may progress to BKPyVAN (15). Early identification of patients at high risk of progression to BKPyV DNAemia and BKPyVAN is clinically relevant, although it is not yet fully understood. There are many potential risk factors for BKPyV-related complications (14), the most significant being the degree of immunosuppression (15). Donor factors include urine BKPyV shedding, high donor pre-transplant BKPyV IgG levels, specific BKPyV genotypes and subgenotypes, donor Human Leukocyte Antigen (HLA)-DQ allele genetic diversity, and donor-recipient genotype mismatch (16–20). Recipient factors include male sex, older age, previous kidney transplantation, use of a ureteral stent, pre-transplant diabetes, episodes of acute rejection, higher HLA mismatch, and obstructive uropathy as the primary renal disease (16, 21–24). Additionally, transplantation-related factors were recently reviewed in a systematic review and meta-analysis. In this study, BKPyVAN was observed with all possible drug combinations without a single identifiable risk factor, suggesting several potential contributing factors and reflecting the complexity of the disease (25).

Although uncommon, BKPyVAN can occur late after transplantation (beyond the first 2 years) and lead to premature allograft loss due to advanced disease at diagnosis (26–28).

In our paper, we present two cases of late-onset BKPyVAN that occurred 9- and 3-years after transplantation, respectively, and provide a review of the underlying factors associated with late-onset BKPyVAN.

## 2 Presentation of two cases

### 2.1 Patient 1

**Figure 1:**
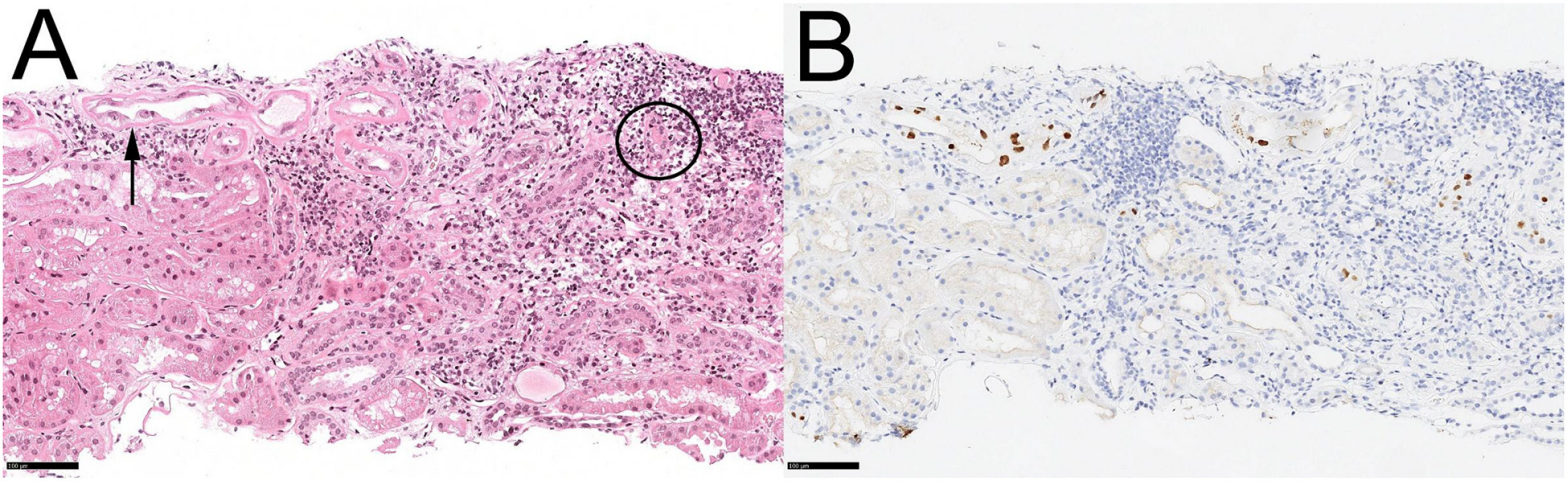
Histopathological findings on indication kidney biopsy: interstitial plasma cell-rich inflammatory infiltrate with areas of moderate lymphocytic infiltration of the tubular epithelium (circled) and apparent nuclear atypia, i.e., vesicular change and coarse clumping in tubular epithelial cell nuclei (arrow), consistent with type 4 viral inclusion bodies of polyomavirus infection. Nascent interstitial fibrosis and early tubular atrophy are also present. [**(A)** HE stain, scale = 100 μm]. Immunohistochemical staining reveals extensive nuclear positivity of tubular epithelial cells for SV-40 T antigen (large T antigen), confirming BKPyVAN [**(B)** scale = 100 μm]. BKPyVAN, BK polyomavirus-associated nephropathy.

**Figure 2:**
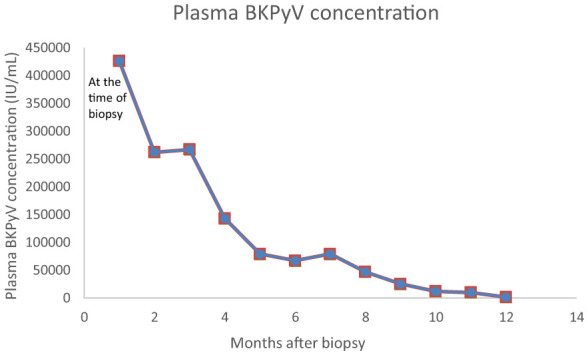
The monthly change in plasma BKPyV concentration in patient 1, measured by qPCR. qPCR, quantitative polymerase chain reaction; BKPyV, BK polyomavirus.

The first patient was a 61-year-old man with end-stage renal failure due to IgA glomerulonephritis who had not received any immunosuppressive treatment before kidney transplantation. He had undergone peritoneal dialysis for 2 years before receiving a successful deceased-donor kidney transplant in the right iliac fossa. As he was not sensitized (virtual panel-reactive antibody [vPRA] level 0%), he was treated with a standard immunological risk immunosuppressive protocol consisting of basiliximab induction, tacrolimus, mycophenolic acid, and methylprednisolone. Because allograft function was immediate and the human leukocyte antigen (HLA)-A, -B, and -DR mismatch was 1,1,1, methylprednisolone was discontinued 5 days after transplantation. Eleven days after transplantation, an indication biopsy was performed due to persistently elevated serum creatinine (SCr) levels of approximately 200 μmol/L (estimated glomerular filtration rate [eGFR], Chronic Kidney Disease Epidemiology Collaboration [CKD-EPI], 30 mL/min/1.73 m^2^). The biopsy revealed acute tubular necrosis and moderate benign nephrosclerosis, with no evidence of early acute rejection. Based on these findings, no additional immunosuppressive therapy was administered, and the patient was discharged on day 14 on dual immunosuppression consisting of extended-release tacrolimus and mycophenolic acid (1,000 mg twice daily). After discharge, the patient was followed regularly at the outpatient clinic, with a gradual decrease in SCr to approximately 150 μmol/L and an eGFR (CKD-EPI) of 45 mL/min/1.73 m^2^. During the first year after transplantation, the patient experienced recurrent drug-induced leukopenia, necessitating administration of granulocyte colony-stimulating factor and a reduction in the daily dose of mycophenolic acid to 1,500 mg (500 mg 3 times daily). BKPyV DNAuria was absent on scheduled urine testing performed every 2 months during the1 year using quantitative polymerase chain reaction (qPCR). A 1-year surveillance kidney biopsy demonstrated weak mesangial staining of IgA in direct immunofluorescence without evidence of rejection or BKPyVAN. The patient continued dual, steroid-free immunosuppression with tacrolimus and mycophenolic acid at the reduced dose (500 mg 3 times daily). The subsequent 7 years after transplantation were uneventful. At routine follow-up visits, the patient remained in good clinical condition with stable allograft function (SCr 160–180 μmol/L; eGFR [CKD-EPI] 35–40 mL/min/1.73 m^2^) and therapeutic tacrolimus trough levels (6–9 ng/mL). He experienced no cardiovascular, infectious, or malignant complications, no rejection episodes, and his remaining medical history was unremarkable. Eight years after transplantation, SCr gradually increased to 245 μmol/L (eGFR [CKD-EPI] 24 mL/min/1.73 m^2^), and urinalysis revealed mild albuminuria (uACR 3.29 g/mol). Kidney ultrasonography showed no abnormalities and demonstrated excellent perfusion of the transplanted kidney. The patient was normotensive and had not recently been prescribed any nephrotoxic medications. Tacrolimus trough levels remained within the therapeutic range (6–7 ng/mL). Donor-derived cell-free DNA (dd-cfDNA) was elevated (1.88%), and donor-specific antibodies (DSA) directed against HLA-A2, HLA-B58, and HLA-DR11 were detected, with mean fluorescence intensities (MFI) of 140, 350, and 540, respectively, as determined by the Luminex single-antigen bead assay. Given suspected rejection, a kidney biopsy was scheduled. As part of the routine pre-biopsy evaluation, BKPyV DNAuria was assessed by qPCR, confirming marked viral shedding at an unquantifiable high concentration (>1,000,000,000 IU/mL; >9 log_10_ IU/mL). Subsequent plasma BKPyV testing by qPCR revealed significant BKPyV DNAemia at 426,000 IU/mL (5.63 log_10_ IU/mL). Histological examination of the kidney biopsy was consistent with sclerosing BKPyVAN, Banff grade 3 (Figure 1), showing characteristic polyomavirus-associated cytopathic changes and positive immunohistochemical staining for Simian Vacuolating Virus 40 (SV40) large T antigen in more than 10% of evaluated tubules. These findings were accompanied by extensive plasma cell–rich interstitial inflammation, foci of moderate tubulitis, and developing interstitial fibrosis and tubular atrophy involving approximately 30% of the biopsy specimen. Immunofluorescence microscopy showed no C4d deposition in peritubular capillaries and no tubular HLA-DR expression. Molecular analysis using the Molecular Microscope Diagnostic System (MMDx) revealed no molecular evidence of rejection. Mycophenolic acid was discontinued, and the daily tacrolimus dose was reduced by 20% (tacrolimus trough level at diagnosis: 6.6 ng/mL). In addition, the patient received intravenous immunoglobulin (IVIG; Octagam, five doses of 0.4 g/kg) to stabilize allograft function and reduce the risk of rejection during the period of minimized immunosuppression. After 2 weeks of low-dose tacrolimus monotherapy, methylprednisolone was introduced at a dose of 4 mg daily to avoid monotherapy. BKPyV DNAemia gradually declined over the subsequent months, reaching 1,400 IU/mL (3,15 log_10_ IU/mL) (Figure 2). Renal function stabilized, with SCr values ranging from 220 to 250 μmol/L (eGFR [CKD-EPI] 23–27 mL/min/1.73 m^2^). At the time of last follow-up (Jan 21, 2026), the patient remains clinically stable on dual immunosuppression with low-dose tacrolimus and methylprednisolone and has experienced no treatment-related complications. Torque teno virus (TTV) DNA was undetectable in plasma in June 2025 and was not assessed thereafter.

### 2.2 Patient 2

**Figure 3:**
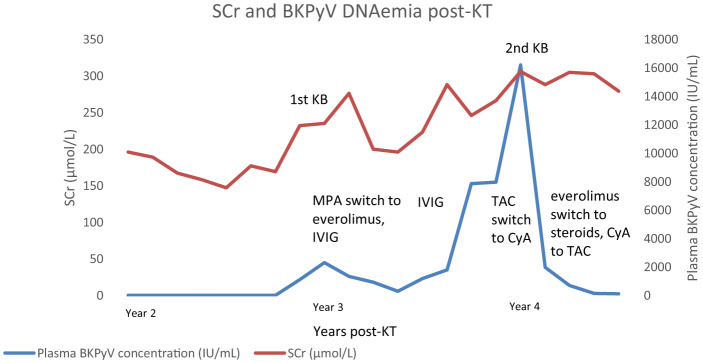
Timeline of SCr and plasma BKPyV DNAemia after kidney transplantation in patient 2. SCr, serum creatinine; BKPyV, BK polyomavirus; KT, kidney transplantation; KB, kidney biopsy; TAC, tacrolimus; CyA, cyclosporine; MPA, mycophenolic acid; IVIG, intravenous immunoglobulins.

The second patient was a 34-year-old man with end-stage renal disease of unknown etiology who underwent a successful kidney transplantation in the right iliac fossa in November 2021. As the patient was not sensitized, he received a standard immunosuppressive regimen consisting of basiliximab induction, tacrolimus, mycophenolic acid, and methylprednisolone. The HLA-A, -B, and -DR mismatch was 1,2,1. Methylprednisolone was discontinued 5 days after transplantation. At discharge on day 10, SCr was 196 μmol/L (eGFR [CKD-EPI] 39 mL/min/1.73 m^2^). Valganciclovir prophylaxis was not initiated because both the donor and the recipient were cytomegalovirus (CMV) seronegative. Over the following weeks, SCr further decreased to 150–180 μmol/L (eGFR [CKD-EPI] 40–50 mL/min/1.73 m^2^). The first year after transplantation was uneventful, and routine urine BKPyV screening by qPCR showed no evidence of viral shedding. A 1-year surveillance biopsy demonstrated no signs of rejection or BKPyVAN, with mild features of calcineurin inhibitor nephrotoxicity. No specific intervention was undertaken, and the patient continued dual, steroid-free immunosuppression with tacrolimus and mycophenolic acid (1,000 mg twice daily). Three years after transplantation, SCr gradually increased to 262 μmol/L (eGFR [CKD-EPI] 27 mL/min/1.73 m^2^). Three weeks before presentation, the patient reported self-limited upper respiratory tract symptoms with low-grade fever, as well as intermittent diarrhea. DSA against HLA-B37, HLA-Cw1, and HLA-Cw6 were detected at low MFI levels (100, 130, and 510, respectively), while dd-cfDNA was mildly elevated at 0.64%. An indication kidney biopsy performed for persistently elevated SCr revealed BKPyVAN, Banff grade II, with positive SV40 immunohistochemical staining and characteristic polyomavirus-associated cytopathic changes in tubular epithelial cells, accompanied by tubulitis, interstitial edema, and mild interstitial fibrosis and tubular atrophy. At the time of biopsy, urine BKPyV concentration was 50,800,000 IU/mL (7.71 log_10_ IU/mL). Plasma BKPyV DNAemia was only mildly elevated at 1,090 IU/mL (3.04 log_10_ IU/mL) but subsequently increased to 2,300 IU/mL (3.36 log_10_ IU/mL). The patient was treated with IVIG (Octagam, five doses of 0.4 g/kg), and mycophenolic acid was replaced with everolimus. Due to persistent diarrhea, the patient underwent colonoscopy with intestinal biopsies. Immunohistochemical staining confirmed CMV colitis. Plasma CMV DNA was detected at a low level of 84 IU/mL (1.92 log_10_ IU/mL), and therapeutic-dose valganciclovir was initiated. Both CMV and BKPyV DNAemia gradually declined, and kidney function stabilized with SCr of 196 μmol/L (eGFR [CKD-EPI] 38 mL/min/1.73 m^2^). In the following months, BKPyV DNAemia decreased further to 273 IU/mL (2.44 log_10_ IU/mL). Valganciclovir was discontinued after 4 months, following clearance of plasma CMV DNAemia and resolution of gastrointestinal symptoms. Six months after the initial diagnostic kidney biopsy, SCr again gradually increased to 288 μmol/L (eGFR [CKD-EPI] 24 mL/min/1.73 m^2^), accompanied by a rise in BKPyV DNAemia to 7,850 IU/mL (3.89 log_10_ IU/mL). The patient was re-treated with IVIG (Octagam, five doses of 0.4 g/kg), and tacrolimus trough levels were reduced to approximately 4 ng/mL. Subsequently, tacrolimus was replaced with cyclosporine when BKPyV DNAemia peaked at 16,200 IU/mL (4.21 log_10_ IU/mL) and SCr increased to 320 μmol/L (eGFR 21 mL/min/1.73 m^2^). At that time, dd-cfDNA was again mildly elevated at 0.69%. A repeat kidney biopsy demonstrated persistent BKPyVAN with extensive interstitial fibrosis and tubular atrophy involving approximately 70% of the biopsy specimen, and no molecular evidence of rejection on analysis using the MMDx. No additional therapeutic interventions were instituted. Following conversion to cyclosporine, BKPyV DNAemia gradually declined to 127 IU/mL (2.10 log_10_ IU/mL). Plasma TTV DNA levels remained below the limit of quantification (< 3.48 log_10_ copies/mL). In the ensuing months, the patient developed lower-extremity edema, and gingival hypertrophy was diagnosed by his dentist. These adverse effects prompted discontinuation of cyclosporine and reintroduction of low-dose tacrolimus. In addition, everolimus was replaced with low-dose methylprednisolone due to progressive allograft dysfunction, peripheral edema, and increasing proteinuria. At the most recent follow-up (Jan 22, 2026), SCr was 259 μmol/L (eGFR [CKD-EPI] 27 mL/min/1.73 m^2^), and BKPyV DNAemia was below the lower limit of quantification (50 IU/mL or 1.70 log_10_ IU/mL). Changes in BKPyV DNAemia and SCr following kidney transplantation are illustrated in Figure 3.

## 3 Discussion

Both of our presented cases highlight that late-onset BKPyVAN can occur even in the absence of intensified immunosuppression and emphasize the importance of considering BKPyV infection in the differential diagnosis of kidney allograft dysfunction, regardless of the time since transplantation or the maintenance immunosuppressive regimen. Recently, a case report was published reporting a kidney transplant recipient who developed late-onset BKPyVAN 15 years post-transplant following chemotherapy for multiple myeloma, including bortezomib, lenalidomide, dexamethasone, and carfilzomib (27). In that case, it was suggested that BKPyVAN late post-transplant should be considered in patients who recently experienced augmented immunosuppression, such as treatment for cancer or rejection (27). Increased incidence of late-onset BKPyVAN was also described in patients with simultaneous kidney-pancreas transplantation, where it was attributed to high-dose immunosuppression and possibly altered immune responses in patients with a history of diabetes (28). In contrast, in our two cases, both non-diabetic patients were on reduced dual immunosuppression when BKPyVAN was diagnosed, highlighting that late-onset BKPyVAN can develop even in the absence of intensified immunosuppression.

A recent systematic review and meta-analysis identified tacrolimus as a major risk factor for BKPyV-associated complications, including biopsy-proven BKPyVAN and BKPyV DNAemia, particularly at higher trough levels (25). Tacrolimus may promote BKPyV replication through renal tubular epithelial injury and stimulation of cellular DNA synthesis (29). Experimental studies suggest that tacrolimus may enhance BKPyV replication via activation of the C3/FN1/NF-κB signaling pathway (29, 31). The combination of tacrolimus and mycophenolate, used in both of our patients, is the immunosuppressive regimen most frequently associated with BKPyV-related complications (30). Although mycophenolate is not considered an independent risk factor, its replacement with everolimus has been proposed as a strategy to suppress BKPyV replication (32, 33). However, results remain inconsistent. In the BKEVER trial, conversion to everolimus did not accelerate viral clearance (25, 34), and experimental evidence suggests that everolimus may directly promote BKPyV activity by increasing TAg expression through inhibition of S-phase kinase-associated protein 2 E3 ligase (35). Consistent with these observations, conversion from mycophenolate to everolimus in our second patient resulted only in a transient decline in BKPyV DNAemia, followed by renewed viral replication despite further reduction of immunosuppression. Steroid-free immunosuppressive regimens may also contribute to BKPyV-related complications, as they often require more intensive use of tacrolimus and mycophenolate. In pediatric kidney transplant recipients, early steroid withdrawal combined with antibody induction, tacrolimus, and mycophenolate has been associated with increased BKPyV risk, especially primary BKPyV infection, although randomized controlled data are lacking (16). Both of our patients received steroid-free immunosuppression and may have experienced primary BKPyV infection, as *de novo* infection could not be distinguished from late viral reactivation.

Other modifiable risk factors for the development of late-onset BKPyV-related complications are kidney re-transplantation, sensitization and formation of *de novo* DSA. Additionally, previous rejection episodes resulting in the inflammation within the allograft may contribute to the development of BKPyV-related complications, which explains why BKPyV almost exclusively affects kidney transplant recipients as opposed to other solid organ transplant recipients (36). Kidney allograft inflammation may also be the result of ischemia-reperfusion injury and promotes similar molecular pathways as in immunosuppressive state (29). Although none of our patients had a history of previous rejection episodes, intensified immunosuppression or delayed graft function, both developed *de novo* DSA at the time of indication biopsy, which may have contributed to the development of BKPyVAN.

Another potential risk factor we observed is recurrent neutropenia, which was present in patient 1 during the first post-transplant year. Although neutropenia may result from BKPyV replication within the bone marrow, even in the absence of detectable BKPyV DNAemia (37), it more commonly reflects excessive immunosuppression and may therefore predispose to uncontrolled viral replication. In such cases, reduction of immunosuppression together with close clinical and virological monitoring is warranted.

Among non-modifiable risk factors, genetic susceptibility has emerged as an important determinant of BKPyV-related complications. Variations in genes regulating innate immune responses, particularly those affecting natural killer (NK) cell function, have been associated with an increased risk of BKPyVAN (38). Individuals carrying fewer activating killer-cell immunoglobulin-like receptors (KIRs) appear to be at higher risk regardless of immunosuppressive burden (39). Similarly, specific HLA alleles acting as KIR ligands have been linked to increased susceptibility, whereas recipient and/or donor expression of HLA-Cw7 and the MICA A5.1 variant may confer protection (40, 41). Although genetic susceptibility was not assessed in our patients, such markers may in the future improve individualized risk stratification, particularly for late-onset BKPyV-related complications beyond the first 2 years after transplantation.

Moreover, with time after transplantation, due to prolonged immunosuppression, there is a gradual decline in BK-virus specific immune responses that may increase the susceptibility to uncontrolled BKPyV replication (26, 42). It seems that T cell inhibition induced by all immunosuppressive agents is the main contributing factor to the development of BKPyVAN (43). In fact, studies have shown that abrogated BKPyV-specific T cell responses are present in patients with BKPyV DNAemia and BKPyVAN, and the recovery of these responses coincides with viral clearance and stabilization of kidney function (44–46). Specifically, the CD4+ T cell response that persists after the clearance of BKPyV DNAemia is a favorable follow-up prognostic factor exhibiting a higher probability of future viral control (47). Monitoring CD4+ T cell response after the resolution of BKPyV DNAemia could aid in individual patients' risk assessment for future BKPyV-related complications. This could have been helpful in case 2, where BKPyV DNAemia could not be easily controlled despite the reduction in immunosuppression. Unfortunately, it was not performed at the time.

Apart from cellular immune responses, humoral immune responses are important in understanding the disease pathology and progression. The most beneficial are the antibodies that neutralize the genotype of the replicating virus, as the presence of neutralizing antibodies against one serotype does not represent protection against a different serotype (48). It was shown that patients with a lower titer of neutralizing antibodies are at risk of developing BKPyV DNAemia. In contrast, patients with broadly neutralizing antibody responses against all BKPyV serotypes are protected against BKPyV DNAemia (48, 49). The value of humoral immune response in fighting against BKPyV in kidney transplant recipients is suggested by the use of IVIG in treating BKPyVAN and potentially in preventing infection in patients with a low neutralizing antibody titer (49). However, the evidence for IVIG use in BKPyV-related complications is mixed and mainly observational. Currently, the use of IVIG is not included in standardized treatment recommendations for BKPyV-related complications and can only be considered as a potential adjunctive therapy (16, 50, 51). A recent systematic review showed that IVIG treatment had no effect on kidney function and could be related to interstitial fibrosis and tubular atrophy (52). In an even more recent pilot randomized controlled trial, the use of IVIG did not show a clear impact on viral clearance, which was associated with higher percentages of BKPyV-specific CD8+ T cells prior to IVIG treatment (0.19% vs. 3.01%; *P* < 0.01) (53). Both our patients were safely treated with IVIG as they may offer immunomodulatory effects to theoretically reduce rejection risk in the time of reduced immunosuppression (16). No benefit was observed regarding BKPyV infection control.

It seems that humoral immunity by itself appears to be insufficient for viral clearance, and cellular immunity is required in controlling and clearing BKPyV infection (54) that can partially be restored by reducing immunosuppression.

Furthermore, changes in the BKPyV genome can occur over time following kidney transplantation (55). Amino acid substitutions in the VP1 region can confer resistance to neutralization by anti-VP1 antibodies, thereby limiting the effectiveness of IVIG therapy (56). Additionally, sequence variations in the NCCR, which serves as the binding site for host transcription factors (TFs), may contribute to viral activation which has been associated with a higher frequency of point mutations, potentially enhancing viral pathogenicity (57). BKPyV-genome alterations are not routinely tested in our center but represent an unmodifiable factor contributing to the complexity of the disease.

Additionally, BKPyV-related complications in kidney transplant recipients may result from primary infection in previously BKPyV-naïve patients, infection with a different viral genotype, or reactivation of latent, recipient-derived BKPyV. The donor kidney has been identified as the primary source of BKPyV both in the early post-transplant period and at later stages (55, 58). BKPyV serotype mismatch between the donor and the recipient is another risk factor for BKPyV-related complications, particularly mismatches involving serotypes Ic and II. Moreover, high ratios of D/R mismatch in neutralizing antibodies against specific serotypes were superior in predicting post-transplant BKPyV DNAemia compared with total antibody response (59). Pre-transplant assessment of neutralizing antibody activity may therefore enable personalized post-transplant risk stratification and inform individualized surveillance strategies.

In both of our cases, the disease likely represented de *novo* BKPyV infection in individuals with impaired immune surveillance and a possible underlying genetic susceptibility. However, neither genetic nor immunological testing was performed, precluding identification of specific predisposing factors. More broadly, predicting late-onset BKPyVAN remains challenging, as most proposed risk factors are still investigational and not routinely incorporated into clinical practice. Furthermore, TTV load, increasingly used as a surrogate marker of the net state of immunosuppression, does not appear to correlate with BKPyV replication (60), a finding also observed in our patients. These observations suggest that BKPyV-specific monitoring, including BKPyV DNAuria, may provide complementary information to TTV load when assessing the adequacy of immunosuppression and the risk of BKPyV-related complications.

Regarding therapeutic strategies, we believe similar approaches should be used as in patients with early BKPyVAN occurring during the first two post-transplant years. Therapeutic management of our patients mostly followed the Second International Consensus Guidelines on the Management of BK Polyomavirus in Kidney Transplantation (16). In case 2, sequential conversion from mycophenolate to everolimus, supported at the time (61), and from tacrolimus to cyclosporine controlled viral replication, although treatment-related adverse effects ultimately necessitated reintroduction of low-dose tacrolimus with methylprednisolone.

In both our patients, although kidney function stabilized following clearance of BKPyV DNAemia, it remained significantly impaired. While late-onset BKPyVAN (occurring more than 2 years post-transplantation) has been described, no dedicated studies have specifically evaluated its prognostic impact relative to early-onset disease. Cohort studies that include late-onset cases (predominantly defined as occurring more than 1 year post-transplantation) have not demonstrated consistent differences in allograft outcomes between these groups (42, 62).

One of the limitations of our cases is the lack of regular BKPyV screening in the second post-transplant year. At the time these two cases were managed, routine BKPyV screening was limited to the first post-transplant year and consisted of qPCR-based detection of BKPyV DNAuria. It therefore remains unclear whether subclinical viral replication may have occurred during the second post-transplant year, although all tests performed within the first year were consistently negative.

Over the past year, our screening strategy has evolved to include annual surveillance beyond the first post-transplant year in all kidney transplant recipients, using urine cytology to enable detection of decoy cells. This extended screening approach may facilitate earlier identification of late-onset BKPyV reactivation and potentially reduce the risk of delayed diagnosis and associated allograft injury.

Finally, given the absence of an effective treatment for BKPyV-related complications, future efforts should increasingly focus on preventive strategies, including vaccination, pre-emptive administration of monoclonal antibodies, or even IVIG (55, 56, 63). At present, these approaches remain investigational and require further clinical validation.

## 4 Conclusion

Late-onset BKPyV-related complications remain a significant diagnostic and therapeutic challenge. BKPyVAN should be considered in the differential diagnosis of allograft dysfunction regardless of time post-transplant or immunosuppressive intensity. Although multiple risk factors have been associated with late-onset disease, their clinical utility remains limited. Early risk stratification is therefore essential but still unresolved, requiring integration of recipient-, donor-, transplant-, immune-, and genetic factors. Future approaches will likely rely on multivariable risk prediction models, potentially supported by artificial intelligence, to enable earlier identification of high-risk patients and guide individualized immunosuppression and surveillance strategies.

## Data Availability

The original contributions presented in this study are included in the article. Further inquiries can be directed to the corresponding author.
